# Development of a surgical procedure for implantation of a prototype suprachoroidal retinal prosthesis

**DOI:** 10.1111/ceo.12287

**Published:** 2014-01-01

**Authors:** Alexia L Saunders, Chris E Williams, Wilson Heriot, Robert Briggs, Jonthan Yeoh, David AX Nayagam, Mark McCombe, Joel Villalobos, Owen Burns, Chi D Luu, Lauren N Ayton, Michelle McPhedran, Nicholas L Opie, Ceara McGowan, Robert K Shepherd, Robyn Guymer, Penelope J Allen

**Affiliations:** 1 Bionics InstituteMelbourne, Victoria, Australia; 2 Centre for Eye Research Australia, The University of Melbourne, Royal Victorian Eye and Ear HospitalMelbourne, Victoria, Australia; 3 Department of Medical Bionics, The University of MelbourneMelbourne, Victoria, Australia; 4 Department of Pathology, Faculty of Medicine, Dentistry and Health Sciences, The University of MelbourneMelbourne, Victoria, Australia

**Keywords:** blindness, cadaver, retinal prosthesis, retinitis pigmentosa

## Abstract

**Background:**

Current surgical techniques for retinal prosthetic implantation require long and complicated surgery, which can increase the risk of complications and adverse outcomes.

**Method:**

The suprachoroidal position is known to be an easier location to access surgically, and so this study aimed to develop a surgical procedure for implanting a prototype suprachoroidal retinal prosthesis. The array implantation procedure was developed in 14 enucleated eyes. A full-thickness scleral incision was made parallel to the intermuscular septum and superotemporal to the lateral rectus muscle. A pocket was created in the suprachoroidal space, and the moulded electrode array was inserted. The scleral incision was closed and scleral anchor point sutured. In 9 of the 14 eyes examined, the device insertion was obstructed by the posterior ciliary neurovascular bundle. Subsequently, the position of this neurovascular bundle in 10 eyes was characterized. Implantation and lead routing procedure was then developed in six human cadavers. The array was tunnelled forward from behind the pinna to the orbit. Next, a lateral canthotomy was made. Lead fixation was established by creating an orbitotomy drilled in the frontal process of the zygomatic bone. The lateral rectus muscle was detached, and implantation was carried out. Finally, pinna to lateral canthus measurements were taken on 61 patients in order to determine optimal lead length.

**Results:**

These results identified potential anatomical obstructions and informed the anatomical fitting of the suprachoroidal retinal prosthesis.

**Conclusion:**

As a result of this work, a straightforward surgical approach for accurate anatomical suprachoroidal array and lead placement was developed for clinical application.

## Introduction

Retinitis pigmentosa (RP) is an inherited retinal degenerative condition that leads to specific loss of photoreceptors and can result in severe vision impairment.^1^,^2^ Some degree of rudimentary vision can be restored in these patients by electrically stimulating the surviving inner retinal neural elements and using the existing visual pathway to create visual percepts (‘phosphenes’).^3^ There are a number of surgical approaches for implanting a stimulating array currently under development. For example, electrode arrays may be placed directly on the surface of the inner retina (epiretinal), under the retina (subretinal), within the suprachoroidal space (suprachoroidal), within the scleral tissue (intrascleral) or on the surface of the sclera (episcleral). With both the epiretinal and subretinal approach, the surgical procedure to place the array may be complicated by trauma to the already diseased retina. The suprachoroidal, intrascleral and episcleral sites are more accessible surgically; however, the array is positioned at a greater distance from the retinal ganglion cells and thus require increased stimulus levels,^4^ which could in turn affect the quality of phosphenes produced. A compromise between a simple, safe and reproducible approach for the implantation of retinal prostheses therefore needs to be weighed against the need to produce high-quality phosphenes from stimulation in order to achieve the ultimate goal of form perception.

Fujikado *et al*. have demonstrated that a visual prosthesis can be safely implanted in a scleral pocket in humans without causing retinal detachment or haemorrhage.^5^ Although the stimulating array was positioned further away from the retinal ganglion cells than the Argus II epiretinal device commercialized by Second Sight Medical Products (Sylmar, CA, USA),^6^ it successfully elicited phosphenes in two patients. As well as having a less invasive surgical procedure than epiretinal implants, the intrascleral device was shown to be stably fixed in the scleral pocket over a 4-week period.^6^ The aim of suprachoroidal implantation was to place the device closer to the retina while maintaining the minimal trauma profile seen in intrascleral implantation.^7^ This approach enables placement of larger arrays to provide a wide field of view, which is beneficial for patient navigation, orientation and mobility.^8^

In order to evaluate the safety and efficacy of a suprachoroidal approach clinically, there is a need for the development of a safe and reliable procedure for human implantation. In particular, the dimensions of the implant need to be refined from those previously used in preclinical studies^7^,^9^,^10^ to suit human anatomy. One of the key issues to address was the variation in axial length of RP patients that has been reported to be between 21.4 and 26.0 mm.^11^

In the present study, we developed the suprachoroidal implantation procedure in cadaver eyes; next, we developed the overall implantation procedure for entire cadavers. This paper summarizes the procedural steps, issues encountered, anatomical landmarks and final dimensions used in our prototype suprachoroidal implant.

## Methods

In study 1A, 14 fixed enucleated eyes (previously used for corneal transplants) were used to develop suprachoroidal implantation procedures, address anatomical obstacles, scleral fixation points, dimensions of the array and fit of the lead exit from the suprachoroidal space to the orbital space. Next, the position of the posterior ciliary neurovascular bundle (PCNB) was characterized in 10 eyes (study 1B). The second study was carried out in six whole cadavers (four fresh and two embalmed) to develop a complete anatomically fitted implantation procedure. This procedure would later be employed in a phase 1 clinical trial (http://www.clinicaltrials.gov, trial # NCT01603576). Study 3 was undertaken to measure the distance from the lateral canthus to pinna (Table 1). This measurement was taken in 61 outpatients at the Royal Victorian Eye and Ear Hospital (RVEEH), Melbourne to gather data on variation among individuals; these data were used to optimize the length of the lead in the clinical device. Ethical approval for all studies was obtained from RVEEH and the University of Melbourne human ethics committees.

**Table 1:** Summary of the studies to investigate surgical issues

Study	Issues investigated
Study 1A	Obstructions to device insertion, adhesions
Enucleated human eye (*n* = 14)	Location of scleral wound
	Relieving incision wound to accommodate axial length and scleral lead exit
	Verify length of array to suit human eye size
	Suture points of scleral patch
	Dimensions of scleral patch
Study 1B	
PNCB measurements (*n* = 10)	Location of PCNB relative to optic disc was investigated
Study 2	Surgical method in humans
Whole human cadavers (*n* = 6)	Develop trocar design and surgical technique
	Lead orbit fixation method
	Location of scleral wound and orbital notch
	Location of scalp percutaneous pedestal wound
	Scleral patch position to allow array position beneath macula
	Angle of lead in orbit
	Suture points of scleral patch
Study 3	Expected variation in lateral canthus to pinna measurements assessed
Lateral canthus to pinna (*n* = 61)	Information gathered informed lead length in the final device

PNCB, posterior ciliary neurovascular bundle.

### Device description

**Figure 1:**
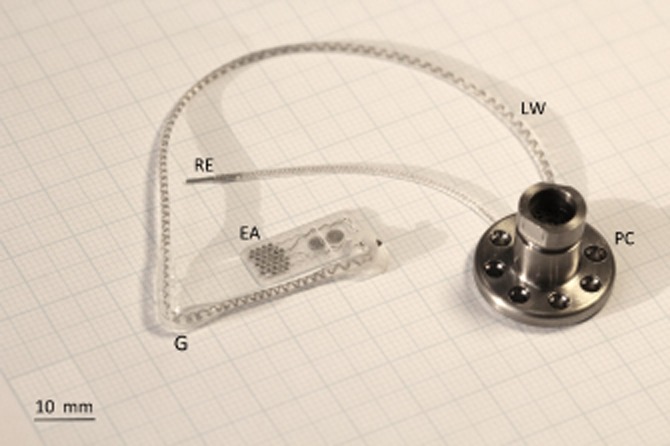
Implantable array and orbital lead wire assembly on the left with percutaneous pedestal on the right. Each abbreviation is as follows: (EA) electrode array, (LW) lead wire, (PC) percutaneous connector, (RE) return electrode and (G) grommet.

Our prototype suprachoroidal retinal prosthesis was composed of a conformable medical grade silicone substrate (dimensions 19 × 8 mm) that houses 21 connected platinum stimulating electrodes. Each electrode was connected via individual insulated platinum/iridium wires bundled to form a helical cable terminating at an external percutaneous pedestal (Fig. 1). The helical cable measures 155 mm in length with an outer diameter of 1.2 mm. A silicone grommet with a cross-sectional width of 2.5 mm was used to anchor the cable at the orbital margin (Fig. 1). All silicone materials are of a medical grade with a long history of biocompatibility.^12^ Implants were designed for implantation in the left eye of a human patient. The retinal prosthesis was originally developed and validated in a feline model.^7^,^13^

### Study 1: enucleated human eye dissection

#### Suprachoroidal implantation

**Figure 2:**
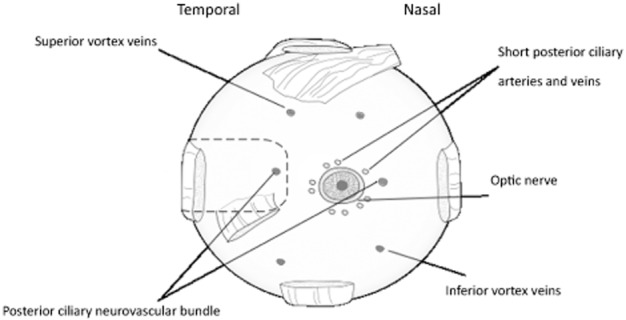
Posterior view of an enucleated eye. The dashed outline shows the ideal anatomical location of the suprachoroidal array pocket. The array tip was advanced posteriorly to beneath the macula yet maintained clearance from the short posterior ciliary vessels.

A 9-mm full-thickness scleral incision was made between 1 and 6 mm superotemporal to the lateral rectus intermuscular septum. With a fixed array length and taking into account the anatomical variations in axial length, the placement of the scleral wound was critical to correctly position the tip of the array beneath the macula. The suprachoroidal space was opened with a crescent blade and dissected with a lens glide (bvi Visitec 581001, Beaver-Visitec International, Inc., Waltham, MA, USA) to create a cleavage plane between the sclera and choroid, and confirm that the PCNB did not traverse across the electrode insertion path. The electrode array was then inserted into the suprachoroidal space. It was necessary to create an ‘L'-shaped wound to position the device. The relieving incision allowed the surgeon to advance the device closer to the optic disc if required. Once the array was in the suprachoroidal space, the lead wire that exits the eye sat proud of the sclera. To allow the lead wire to sit flush with the sclera, a small triangular wedge of sclera was removed at the superior margin to reduce the height of the protruding lead. The scleral incision was closed with 8/0 nylon sutures, and a scleral anchor patch was sutured onto the sclera with 8/0 nylon sutures. To check the position of the retinal implant in this cadaver study, the eye was dissected and the array position confirmed. The preferred anatomical array position beneath the macula is shown in Figure 2.

#### Scleral patch and suture points

**Figure 3:**
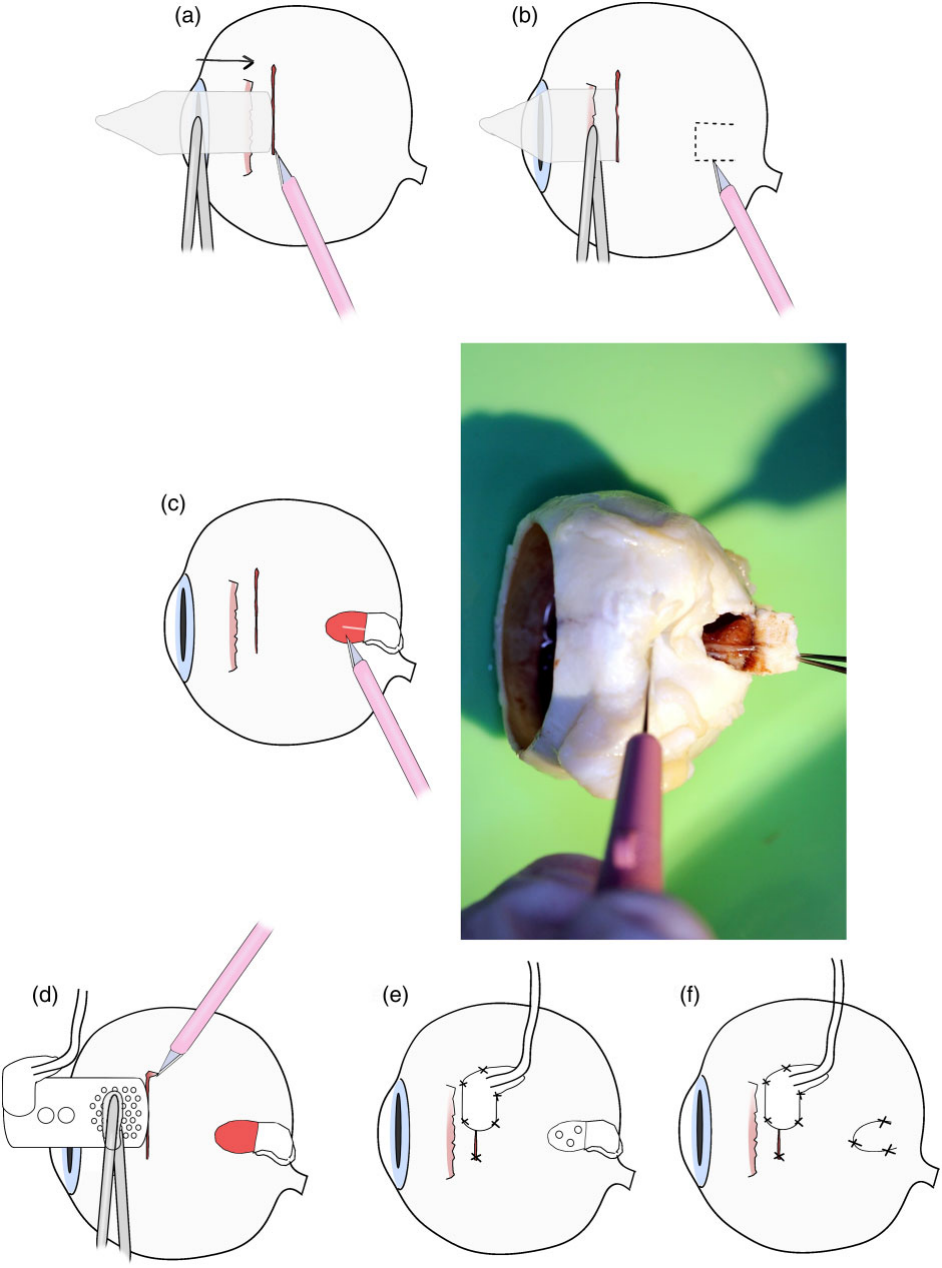
Procedure to address the issue of posterior ciliary neurovascular bundle (PCNB) obstruction during array insertion. (a) First, a 9-mm full-thickness scleral incision was made, (b) then the suprachoroidal space was probed with a lens glide to find any potential obstruction. (c) If an obstruction was present, a ‘trap door’ scleral incision of 2 × 2 mm was created, and the PCNB was severed.(d) The array was then inserted into the suprachoroidal space. (e) The scleral wound and patch were then sutured. (f) Finally, the ‘trap door wound’ was sutured.

A scleral patch attached to the lead wire consisted of Dacron coated in medical grade silicone. This patch provided a scleral lead fixation point that allowed eye movement while the implanted array was kept in a stable suprachoroidal position. Nylon 8/0 interrupted sutures were used to secure the patch to the sclera (Fig. 3e).

#### Anatomical obstructions during insertion of the electrode array

Fourteen fixed enucleated human eyes were used (study 1A) to investigate the PCNB, which in nine eyes traversed the intended array position in the suprachoroidal space. To address this obstruction, a ‘trap door’ procedure was developed (Fig. 3c). A scleral incision was made as previously described and a lens glide used to extend the suprachoroidal space. A full scleral thickness trap door was created posterior to the lateral rectus muscle. The wound provided access to ablate the PCNB within the suprachoroidal space, allowing the device to be advanced to an optimal position beneath the macula. The trap door wound would be sutured, and the previously described suprachoroidal implantation procedure was used (Fig. 3). To further investigate the anatomical variation in PCNB location, the distance that incorporated curvature of the eye from centre of the optic disc to PCNB was measured in 10 defrosted enucleated eyes (study 1B).

### Study 2: surgical procedure in human cadavers

#### Percutaneous pedestal

**Figure 4:**
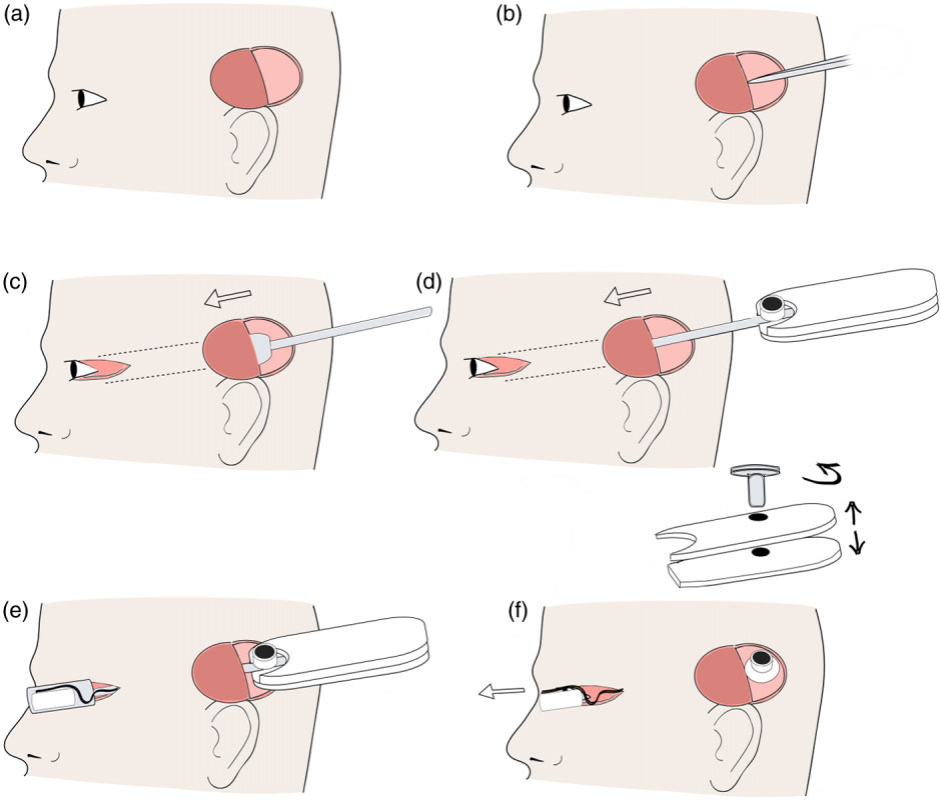
Pedestal placement and tunnelling. (a) First, an incision site superior to the pinna was made and a full thickness ‘C’ shape flap created on the scalp. (b) Incision was made in temporalis facia, and a tunnel was created between muscle and facia. (c) The tunnelling trocar was passed anterior from ‘C’ shape flap to the lateral canthus to create a tunnel. The temporalis facia was incised lateral to the orbit margin to allow the trocar to pass. (d) The array was placed in the trocar and tunnelled from pinna to the lateral canthus. (e) The lid of trocar was removed. (f) The handle screw was removed, and the handle was separated. The array was then removed from trocar, and the trocar was removed from the head through the lateral canthus wound.

Using a full cadaver enabled the surgeons to develop the lead wire routing, practice the percutaneous connector implantation and optimize orbital access procedures. A full-scale dummy retinal implant was placed on the skin between the pinna and orbital margin. The location of the percutaneous pedestal and position of ‘C’ shape incision was marked out posterior to the pinna^14^ (Fig. 4). A ‘C’ shape full-thickness skin incision was made and skin flap was retracted to expose the temporalis fascia and periosteum (Fig. 4a). The periosteum was incised and elevated to create a seat for the percutaneous pedestal. The device was tunnelled forward, as described in implant tunnelling. To secure the pedestal to the skull four 7 mm titanium (91–6207 2.0 × 7 mm) self-drilling, self-tapping Biomet screws (Microfixation, Inc., Jacksonville, FL, USA) were used. Additional screw holes in the pedestal were available if needed. All screws held securely during this study, however, without the need for additional holes.

#### Implant tunnelling

**Figure 5:**
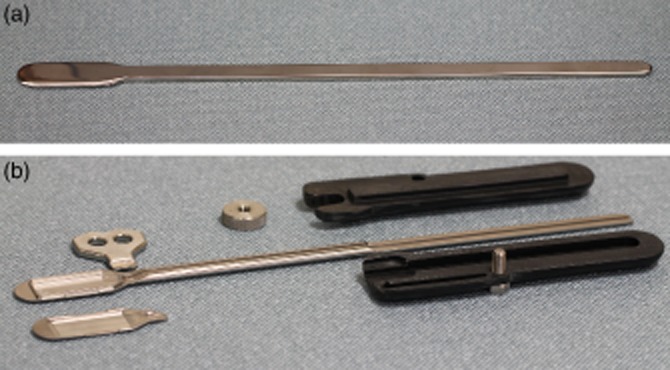
Image of the custom trocar for safely tunnelling the device from the ‘C’ incision close to the pinna to the lateral canthus. (a) The trocar used to create the first tunnel. (b) The trocar that housed the device during insertion. On the left is the housing where the array was clamped in place. The black handle screws together to hold the pedestal in place during implantation.

Two custom trocars were developed to create a tunnel beneath temporalis fascia and safely deliver the helical cable and retinal electrode array to the orbit. The first solid tunnelling trocar was designed to create a tunnel to allow safe passage of the second trocar (Fig. 5a). Second, the implant trocar was used to pass the array through the tunnel from the temporoparietal ‘C’ incision to the orbital margin (Fig. 4e). The implant trocar incorporated a customized head cavity to house the electrode array. It also had a grooved arm and detachable handle to stabilize the helical cable and percutaneous pedestal during the procedure (Fig. 5b). The temporalis fascia was incised anterior to the site for the percutaneous pedestal, and then a plane was created between the temporalis muscle and temporalis fascia using blunt dissection. The tunnel was extended forwards to the orbital margin, keeping deep to temporalis fascia to protect the frontal branch of the facial nerve. The tunnel was widened using the trocar, and the temporalis fascia was incised lateral to the orbital margin via the lateral canthotomy to allow the trocar to pass. The electrode array and lead wire assembly were loaded into the implant trocar and passed forward through the temporalis tunnel. Once the head of the trocar (and retinal implant) cleared the orbital margin, the trocar lid and detachable handle were removed. Because of its streamlined nature, the trocar could then be passed forward and removed through the canthotomy incision. In this way, tissue trauma was kept to a minimum, and the lead wire was ideally situated (Fig. 4f).

**Figure 6:**
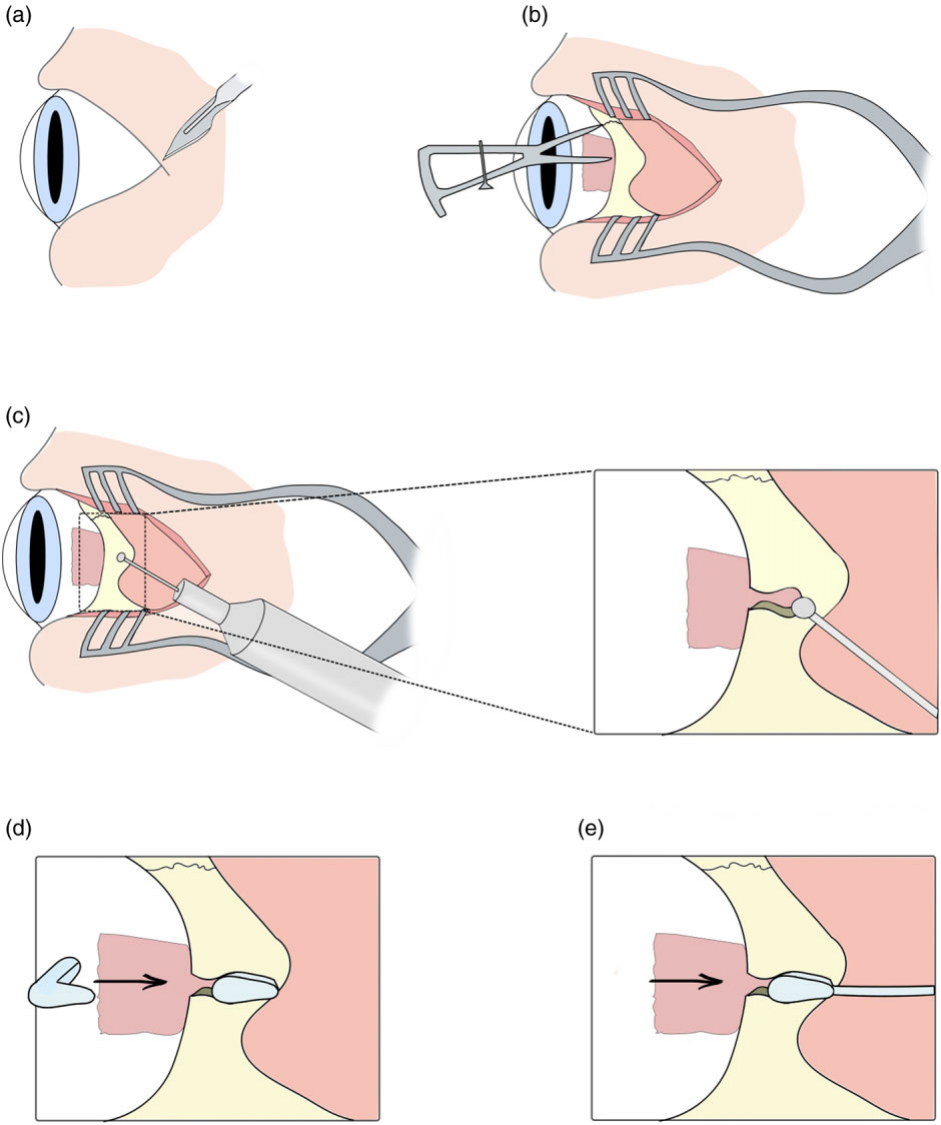
Orbitotomy for the placement of orbit grommet and lead. (a) First, a lateral canthus was created. (b) The orbital zygomatic bone was exposed, and 10–12 mm inferior of zygomaticofrontal suture line was measured using callipers. (c) An orbitotomy was created using a Bien Air drill with a 1.8 mm cutting burr and undercut with 2.3 mm cutting burr. (d) The fit was checked with a silicone dummy orbit grommet. (e) The dummy grommet was removed, and then the orbital grommet and lead were inserted into the orbitotomy.

For stabilization of the lead wire, an orbitotomy was created 10–12 mm below the zygomaticofrontal suture. The orbitotomy provided an anchor point for the lead wire. A compression fit of the orbital grommet was used to secure the lead wire without crushing it (Fig. 6e). Protruding silicone lugs on the grommet assisted in securing the grommet in place. The width of the grommet to be placed in the orbital margin was 2.5 mm. Using a cutting burr, a notch was made in the orbital margin to house the grommet. Various notch configurations were tested to determine the most secure fit for the grommet. A two-stage approach was adopted to create an ‘over-hanging’ cut. A 1.8 mm burr was used to create the initial orbitotomy followed by a 2.3 mm burr used to undercut the notch to prevent crushing of the cable and creating a more secure anchor point (Fig. 6). A dummy orbital silicone grommet was used to test the notched bone to confirm a tight fit. The lead grommet was placed into the zygomatic bone once the suprachoroidal implantation was complete. The periosteum was closed over the orbitotomy with 6/0 vicryl sutures to secure the grommet in place. The position of the lead was inspected visually, and forced duction tests were performed to verify normal range of eye motion. A skin incision between the orbit and pinna was made to confirm the lead was over the temporalis muscle and under the temporalis facia. Finally, the return electrode (Fig. 1) was placed in a subperiosteal pocket beneath temporalis muscle inferior to the percutaneous pedestal.

#### Suprachoroidal implantation

**Figure 7:**
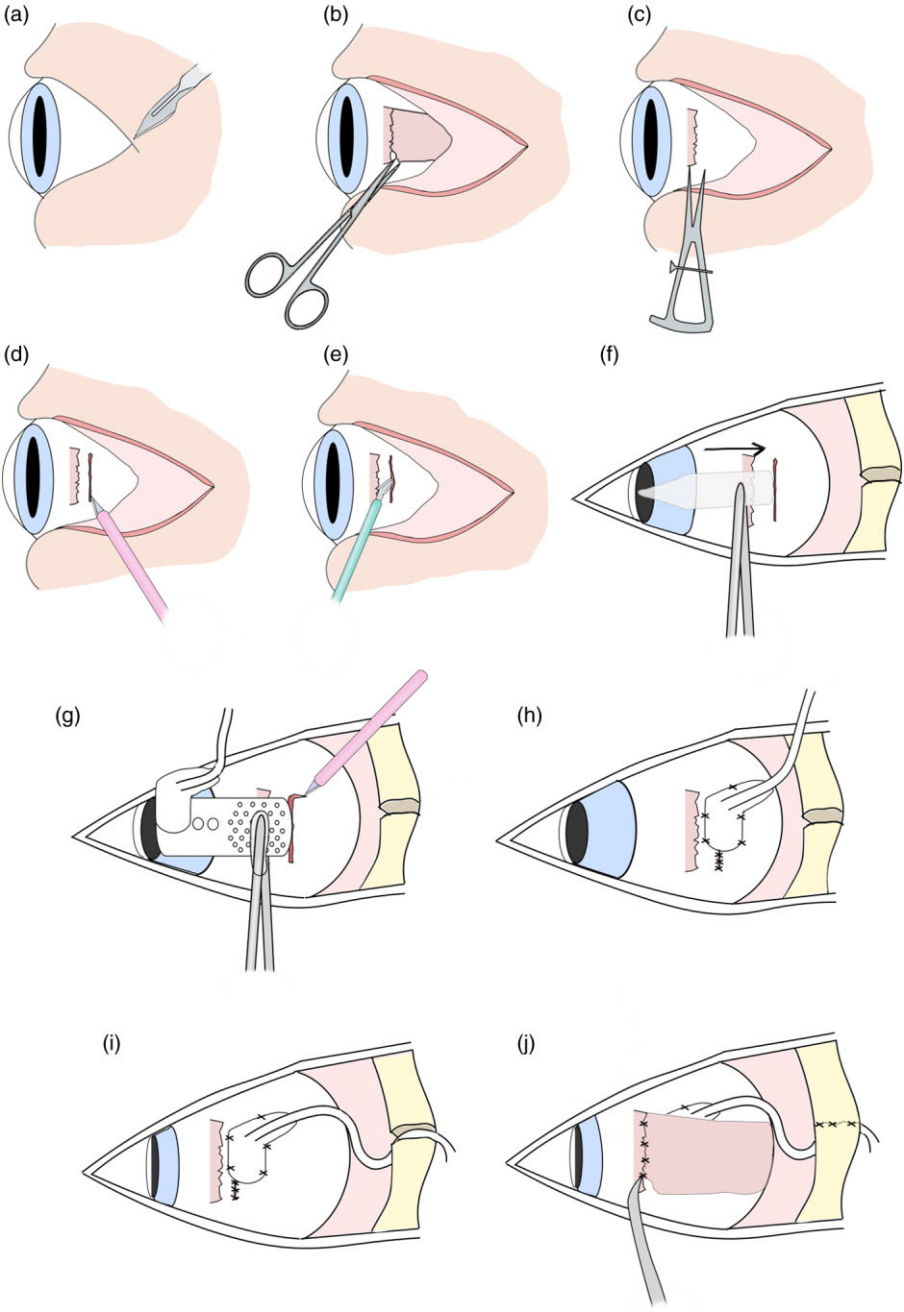
Surgical implantation of suprachoroidal retinal prosthesis in cadavers. (a) First a lateral canthotomy was created. (b) The lateral rectus muscle was detached. (c) Scleral wound placement was measured 1–2 mm posterior to lateral rectus muscle. (d) A 9-mm full-thickness scleral incision was created. (e) A pocket was made in the suprachoroidal space using a crescent blade. (f) The pocket was then probed with a lens glide to extend the space. (g) Prosthesis was inserted and then a relieving incision made to adjust placement. (h) Sclera and scleral patch were sutured using interrupted sutures. (i) The lead was then routed 15–30° superior to wound, and the orbit grommet was inserted into the orbitotomy. (j) Finally, the lateral rectus muscle was reattached with 6/0 vicryl sutures and the periosteum sutured.

The suprachoroidal implantation method that was developed in enucleated eyes in study 1 was then refined in whole cadavers in study 2 (Fig. 7). To gain access to the sclera to make an incision, a lateral canthotomy approximately 15 mm in length was made. The lateral rectus muscle was ligated with 6/0 vicryl sutures and detached. A 9-mm full-thickness scleral incision was made 1–2 mm posterior to the lateral rectus intermuscular septum. The suprachoroidal space was opened with a crescent blade and probed with a lens glide. After placement of the array in the suprachoroidal space and closure of the scleral wound, the lateral rectus muscle was reattached (Fig. 7j).

### Study 3: characterization of extraorbital of lead length

To determine the required lead wire length, a study was completed on 61 adult outpatients (33 men and 28 women). One examiner took three repeated measures of the distance between the pinna and lateral canthus in these patients. These measurements were used to find the mean, standard deviation and range of the pinna to lateral canthus dimensions.

## Results

### Scleral wound position

Variation in ocular axial length was the primary factor to address in order to ensure that the array was placed beneath the macula and was further than 2 mm away from the optic disc to minimize trauma.^7^ The two approaches to tackle this issue were the position of scleral wound and use of a relieving incision. In enucleated eyes, measuring the distance between lateral rectus intermuscular septum to the lateral margin of the optic nerve head provided a reference point to estimate where to place the incision for the 19 mm long electrode array. In study 1 (enucleated eyes), the 9-mm scleral incision was placed between 1 and 6 mm superotemporal to the lateral rectus intermuscular septum, and in study 2, it was placed 1–2 mm posterior to the lateral rectus muscle septum. As the scleral wound is sutured beneath the scleral patch, minimal size and flexibility of the patch were paramount. From these studies, the dimensions of the scleral patch were refined to 4 × 7 mm.

### PCNB

**Figure 8:**
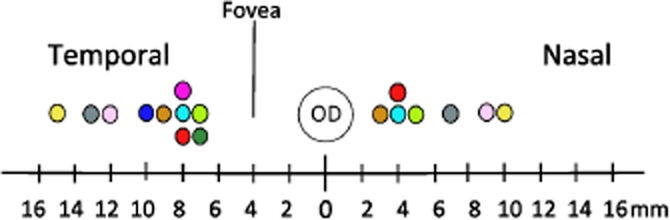
Distance from centre of the optic disc (OD) to posterior ciliary neurovascular bundle (PCNB) was measured on the nasal and temporal side; approximate distance of fovea to OD is indicated. Each colour represents an individual (*n* = 10). The centre circle represents the OD, and coloured circles are the distance including curvature of the eye from PCNB to OD (mm).

In 9 out of the 14 eyes examined, the insertion path was obstructed by the PCNB. Distance from the optic disc to where the PCNB traversed the space varied. A technique was developed to ablate the bundle if detected after probing. The sclera above the PCNB was surgically accessible once the lateral muscle was detached. The trap door procedure of cutting a 2 to 3 mm scleral flap allowed the PCNB to be severed (Fig. 3). Severing of the PCNB allowed unimpeded insertion of the electrode array. Measurements from centre of the optic disc to the PCNB in study 1B confirmed that an array inserted in the temporal side would be obstructed. Temporal PCNB position to the centre of the optic disc ranged from 7 to 15 mm (Fig. 8). In patients, the PNCB would be ablated using diathermy.

### Lead routing

The surgical method of creating a ‘C’ shape flap and securing the percutaneous pedestal to the skull was previously developed for use in cochlear implant surgeries.^14^ The retinal prosthesis has a delicate intraocular array, however, which needed to be tunnelled from behind the pinna to the eye, requiring the development of a custom trocar. The trocar enabled the electrode array to be passed beneath the temporalis facia without causing mechanical damage to the array with minimal risk of trauma. This was achieved by the contoured form of the trocar and incorporation of a detachable handle allowing the trocar to slide through the tunnel for removal (Figs 5).

A number of anatomical constraints had to be overcome when routing the lead from the eye to the pinna. There needed to be sufficient slack on the lead to allow the full range of eye movement while avoiding twisting or damage to the lead. An orbital lead exit angle of 15–30° superior to the scleral wound and 34-mm orbital lead provided a gradual curve between the eye and the zygomatic bone. A forced duction test was used to confirm full range of eye movement with the orbital lead attached.

A tight-fitting orbitotomy was essential to provide mechanical stability and minimize the likelihood of extrusion around the zygomatic bone. A dummy orbital grommet was used to confirm the size of the orbitotomy. This minimized unnecessary handling of the delicate electrode array. The orbitotomy allowed a 2.5-mm diameter grommet to be firmly seated in the orbitotomy without crushing the lead (Fig. 6). The lateral canthus-to-pinna distance was measured in 61 outpatients at the RVEEH. The average distance was 80 ± 1 mm with a range of 70–92 mm. The total lead length from array to pedestal including the intraorbital loop was 155 mm.

## Discussion

With the use of enucleated eyes and cadavers, a safe and effective surgical technique for the implantation of a prototype suprachoroidal electrode array and lead wire assembly was developed. The implantation procedure was adapted to suit individual anatomical variations in humans, including axial length and pinna to canthus length. This allowed the device to be delivered to the desired location beneath the macula. The grommet was positioned in a mechanically stable position through a compression fit orbitotomy, and suturing of the contoured scleral patch allowed free eye movement while providing a fixation point for the lead wire. Stable fixation is critical to reduce migration, fibrosis and micromovements of the device, which may result in unreliable phosphenes in patients.

Axial length variation between individuals was one of the most critical anatomical issues to address. In enucleated eyes, the distance from the lateral rectus muscle to the optic disc was measured to estimate wound position relative to lateral rectus muscle. In cadavers, it was not possible to measure because of lack of access to the posterior orbit, and hence, the wound position did not vary to such an extent. In live patients, it is possible to measure the axial length preoperatively and obtain good-quality imaging of the orbit, globe and retina to confirm optimal wound position. In the clinical phase I trial that followed this development work, all three patients had successful electrode placement and wound healing utilizing these techniques.^15^

It is vital that the inserted array does not impinge on the optic disc and surrounding short posterior ciliary vessels during insertion, as this would result in immediate and devastating optic nerve damage or suprachoroidal haemorrhage with subsequent vision loss.^7^,^16^ The relieving incision at the superior end of the wound allowed the array to be further advanced under visual guidance if required, allowing a slow and measured advance towards the macula. A small section excised from of the sclera at the superior anterior margin minimized rotation of the array upon insertion and allowed the lead wire to sit flat on the sclera. Wound position evolved from parallel to the intermuscular septum and superotemporal to the lateral rectus muscle to posterior to the lateral rectus muscle. The former position had the advantage of thicker sclera, whereas the latter provided support for scleral wound closure. Individual variation in orbit size would impact on how the lead sits within the orbit. A smooth intraorbital loop was achieved with lead angle adjusted from 15 to 30° superior.

Individual variations in the location where the PCNB traversed the sclera had the potential to limit the number of suitable individuals for suprachoroidal implantation. The idea of angling the array to avoid the PCNB was briefly explored. However, other vascular structures that traverse the suprachoroidal space such as the vortex veins would obstruct the device insertion (data not shown). Ablating the PCNB provided a viable option because the area was accessible during surgery; however, the clinical effects are unclear. Occlusion of the posterior ciliary artery was investigated in Rhesus monkeys that showed a temporary vascular filling defect. One to 2 days later, vascular filling started to improve.^17^ In the absence of a method to visualize where the PCNB traverses the sclera, surgeons need to be prepared to carry out the trap door procedure once the suprachoroidal space was probed and an obstruction was discovered. Although the PCNB had the potential to obstruct array insertion, to date, it has not posed an issue in clinical trials (*n* = 3).^15^ However, numbers were too small to draw any final conclusions about PNCB obstructions in patients. One possibility of the PCNB not obstructing the array insertion may be due to vessel mobility. Fixed tissue used for the array insertion was much stiffer than living tissue, which was a limitation with the cadaver studies.

Accurate measurements of the distance from the lateral canthus to the pinna were also critical to reduce excess lead length. Minimizing lead length prevented the lead looping over itself and therefore was beneficial in reducing the chance of extrusion. Routing the lead through the orbitotomy also minimized the chance of extrusion by securely holding the lead in place. The compression fit and suturing the pereostium over the orbitotomy meant the use of bone cement as a means of fixing the lead wire at the orbit were not necessary.

During the surgical development, a novel trocar was manufactured to protect the electrode array and cable. Secure housing of the implant was paramount for passage of the array through the temporalis tunnel to the lateral canthus. The detachable handle prevented movement of the percutaneous pedestal at the distal end. The cavity and lid housed the array in place at the proximal end (Fig. 5b).

To date, clinical trials of retinal prostheses have typically used either epiretinal or subretinal surgical approaches. Argus II, implanted epiretinally, has shown improvements in orientation, mobility and spatial locomotion in people with end-stage RP.^5^ Improvements in orientation tasks have also been demonstrated with the subretinal device from Retina Implant AG.^18^ However, these surgical approaches are not without complications. Stable fixation of an epiretinal device has been an issue, and in some cases, the device had to be retacked postimplantation. Reported serious adverse events such as conjunctival erosion and endophthalmitis indicate a need for a simpler and safer surgical procedure.^6^,^19^ Intrascleral implantation has had a promising safety profile in the two implanted patients but is not ideal because of the large distance between electrodes and retinal ganglion cells.^5^

The surgical approach for most retinal prosthesis involves long and complicated surgery. For example, subretinal implants often require 6 to 8 h ocular surgery under general anaesthesia.^20^ Benefits of a simplified surgical approach would be favourable to both prosthesis recipients and surgeons. Although cadaver work is ideal for surgical development, it is limited in two main areas: cadavers do not bleed, and it is not possible to accurately measure axial length in eyes with corneal opacification. This reported novel suprachoroidal surgical procedure allows stable fixation of the device and lead, which is of critical importance for effective retinal stimulation. As a result of this human cadaver work, a safe and effective surgical approach for accurate anatomical suprachoroidal array placement, lead placement and safe tunnelling, has been developed for clinical use, which will be of vital importance in future clinical trials.

## References

[1] Daiger SP, Bowne SJ, Sullivan LS (2007). Perspective on genes and mutations causing retinitis pigmentosa. Arch Ophthalmol.

[2] Hartong DT, Berson EL, Dryja T (2006). Retinitis pigmentosa. Lancet.

[3] Shepherd RK, Shivdasani MN, Nayagam DA, Williams CE, Blamey PJ (2013). Visual prostheses for the blind. Trends Biotechnol.

[4] Balthasar CD, Patel S, Roy A Factors affecting perceptual thresholds in epiretinal prostheses. Invest Ophthalmol Vis Sci.

[5] Fujikado T, Kamei M, Sakaguchi H Testing of semichronically implanted retinal prosthesis by suprachoroidal-transretinal stimulation in patients with retinitis pigmentosa. Invest Ophthalmol Vis Sci.

[6] Humayun MS, Dorn JD, da Cruz L (2008). Interim results from the international trial of second sight's visual prosthesis. Ophthalmology.

[7] Villalobos J, Allen PJ, McCombe MF (2012). Development of a surgical approach for a wide-view suprachoroidal retinal prosthesis: evaluation of implantation trauma. Graefes Arch Clin Exp Ophthalmol.

[8] Curriero FC, Pinchoff J, van Landingham SW, Ferrucci L, Friedman DS, Ramulu PY (2013). Alteration of travel patterns with vision loss from glaucoma and macular degeneration. JAMA Ophthalmol.

[9] Villalobos J, Nayagam DA, Allen PJ (2012). A wide-field suprachoroidal retinal prosthesis is stable and well tolerated following chronic implantation. Invest Ophthalmol Vis Sci.

[10] Wong YT, Chen SC, Seo JM, Morley JW, Lovell NH, Suaning GJ (2009). Focal activation of the feline retina via a suprachoroidal electrode array. Vis Res.

[11] Walia S, Fishman GA (2008). Retinal nerve fiber layer analysis in RP patients using Fourier-domain OCT. Invest Ophthalmol Vis Sci.

[12] Habal MB (1984). The biologic basis for the clinical application of the silicones a correlate to their biocompatibility. Arch Surg.

[13] Shivdasani MN, Luu CD, Cicione R (2010). Evaluation of stimulus parameters and electrode geometry for an effective suprachoroidal retinal prosthesis. J Neural Eng.

[14] Van den Honert C, Kelsall DC (2007). Focused intracochlear electric stimulation with phased array channels. J Acoust Soc Am.

[15] Allen PJ, Yeoh J, McCombe M (2013).

[16] Izci Y, Gonul E (2006). The microsurgical anatomy of the ciliary ganglion and its clinical importance in orbital traumas: an anatomic study. Minim Invasive Neurosurg.

[17] Hayreh SS, Baines JAB (1972). Occlusion of the posterior ciliary artery. Br J Ophthalmol.

[18] Wilke R, Gabel VP, Sachs H (2011). Spatial resolution and perception of patterns mediated by a subretinal 16-electrode array in patients blinded by hereditary retinal dystrophies. Invest Ophthalmol Vis Sci.

[19] Ahuja AK, Behrend MR (2013). The Argus™ II retinal prosthesis: factors affecting patient selection for implantation. Prog Retin Eye Res.

[20] Stingl K, Bach M, Bartz-Schmidt KU (2013). Safety and efficacy of subretinal visual implants in humans: methodological aspects. Clin Exp Optom.

